# Supplementary material for: Drug repurposing for aging research using model organisms
Source: Aging Cell. 2017 Jun 16;16(5):1006–15. doi: 10.1111/acel.12626 (PMC5595691; doi:10.1111/acel.12626)
Supplement: Supplementary file 7 — Data S1 Zip‐Archive of all report cards. [file ACEL-16-1006-s007.zip › RC_CMP.pdf]

CMP

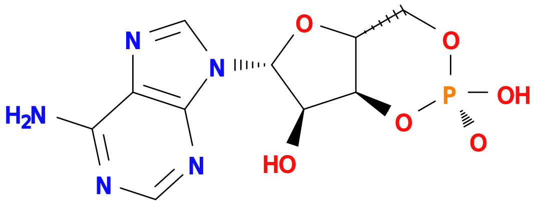

Database identifiers

ChEMBLCompound	CHEMBL316966
CHEBI	17489
eMolecules	474950

Ranking

	Rank	Score
Drosophila	291/697	0.572
C. elegans	NA	NA

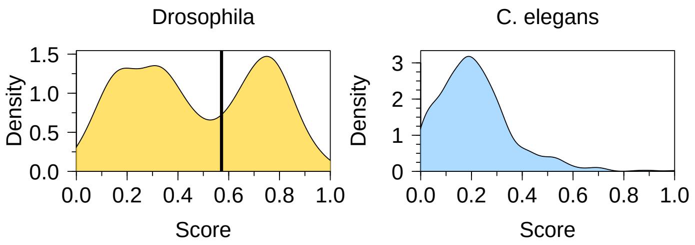

	Ageing implication	Domain conservation	Binding site conservation	Binding affinity	Bioavailability	Lipinski	Promiscuity	Purchasability	Drug approval	**Total**
Drosophila	1.0	0.958	0.885	0.715	(0.9)	-0.05	-0.024	0.1	0.0	0.572
C. elegans	NA	NA	NA	NA	NA	NA	NA	NA	NA	NA

Names

- **3',5'-cyclic AMP**
- Adenosine 3',5'-cyclic phosphate
- Adenosine 3',5'-phosphate
- Adenosine-3',5'-cyclic-monophosphate
- cAMP
- Cyclic Adenosine Monophosphate
- Cyclic adenylic acid

Roles

human metabolite

Status

Approved drug (according to ChEMBL)	No
Number of Rule of 5 violations	1
Binding affinity to original target in log units (RF-Score prediction)	5.92
Burns *C. elegans* bioavailability prediction	-5.75

Compound Target Characteristics

Potassium/sodium hyperpolarization-activated cyclic nucleotide-gated channel 2

Best gene implication in ageing for this target family came from gene Q8IGX2 annotated in UniProt release 2014_02. Annotation GO 8340 (determination of adult lifespan) was Inferred from Mutant Phenotype

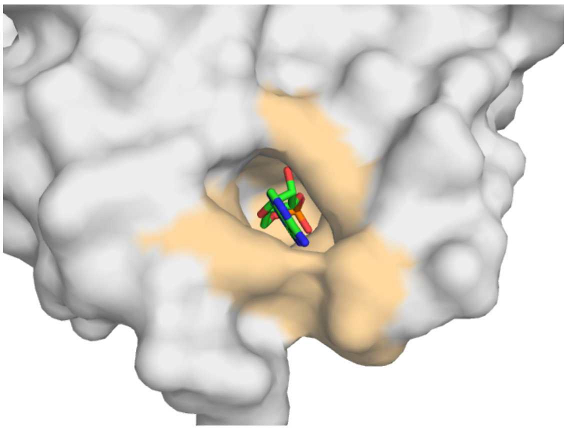

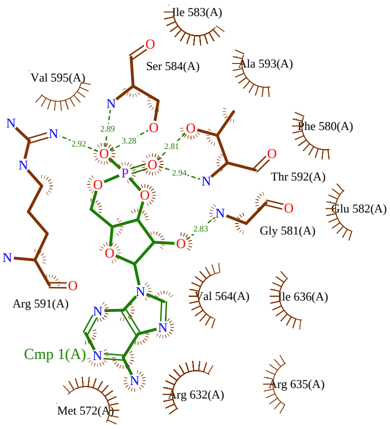

protein	amino acids contacts (binding site)														
PDB:1q43:chainA:O88703	I	V	M	F	G	E	I	C	R	T	A	V	R	R	-
PDB:1q5o:chainA:O88703	I	V	M	F	G	E	I	C	R	T	A	V	R	R	I
PDB:3bpz:chainA:O88703	I	V	M	F	G	E	I	C	R	T	A	V	R	R	I
PDB:3etq:chainA:O88703	I	V	M	F	G	E	I	S	R	T	A	V	R	R	I
sp:Q9UL51:HCN2_HUMAN	I	V	M	F	G	E	I	C	R	T	A	V	R	R	I
sp:O60741:HCN1_HUMAN	I	V	M	F	G	E	I	C	R	T	A	V	R	R	I
tr:F1LRY7:F1LRY7_RAT	I	V	M	F	G	E	I	C	R	T	A	V	R	R	I
tr:F1LSH6:F1LSH6_RAT	I	V	M	F	G	E	I	C	R	T	A	V	R	R	I
sp:O88703:HCN2_MOUSE	I	V	M	F	G	E	I	C	R	T	A	V	R	R	I
sp:O88704:HCN1_MOUSE	I	V	M	F	G	E	I	C	R	T	A	V	R	R	I
tr:Q8IGX2:Q8IGX2_DROME	I	V	T	F	G	E	I	C	R	V	A	V	R	K	I
tr:A1Z9P0:A1Z9P0_DROME	I	V	T	F	G	E	I	C	R	V	A	V	R	K	I
tr:Q56JH9:Q56JH9_DROME	I	V	T	F	G	E	I	C	R	V	A	V	R	K	I
tr:F7VJU3:F7VJU3_DROME	I	V	T	F	G	E	I	C	R	V	A	V	R	K	I
tr:Q56JH8:Q56JH8_DROME	I	V	T	F	G	E	I	C	R	V	A	V	R	K	I
tr:Q9Y1J9:Q9Y1J9_DROME	I	V	T	F	G	E	I	C	R	V	A	V	R	K	I
tr:Q56JI0:Q56JI0_DROME	I	V	T	F	G	E	I	C	R	V	A	V	R	K	I
tr:B7YZE7:B7YZE7_DROME	I	V	T	F	G	E	I	C	R	V	A	V	R	K	I
tr:A1Z9N8:A1Z9N8_DROME	I	V	T	F	G	E	I	C	R	V	A	V	R	K	I
tr:Q56JH7:Q56JH7_DROME	I	V	T	F	G	E	I	C	R	V	A	V	R	K	I
tr:B7YZE8:B7YZE8_DROME	I	V	T	F	G	E	I	C	R	V	A	V	R	K	I
tr:A1Z9N7:A1Z9N7_DROME	I	V	T	F	G	E	I	C	R	V	A	V	R	K	I

protein	whole protein ident	whole protein simil	domain-based ident	domain-based simil	contact-based ident	contact-based simil
PDB:1q43:chainA:O88703	1.0	1.0	1.0	1.0	0.93	0.86
PDB:1q5o:chainA:O88703	1.0	1.0	1.0	1.0	1.0	1.0
PDB:3bpz:chainA:O88703	0.99	1.0	0.99	1.0	1.0	1.0
PDB:3etq:chainA:O88703	0.98	0.99	0.98	0.99	0.93	0.93
sp:Q9UL51:HCN2_HUMAN	0.91	0.95	1.0	1.0	1.0	1.0
sp:O60741:HCN1_HUMAN	0.57	0.74	0.91	0.98	1.0	1.0
tr:F1LRY7:F1LRY7_RAT	0.96	0.96	1.0	1.0	1.0	1.0
tr:F1LSH6:F1LSH6_RAT	0.55	0.71	0.91	0.98	1.0	1.0
sp:O88703:HCN2_MOUSE	1.0	1.0	1.0	1.0	1.0	1.0
sp:O88704:HCN1_MOUSE	0.55	0.71	0.91	0.98	1.0	1.0
tr:Q8IGX2:Q8IGX2_DROME	0.26	0.38	0.67	0.91	0.8	0.88
tr:A1Z9P0:A1Z9P0_DROME	0.38	0.58	0.67	0.91	0.8	0.88
tr:Q56JH9:Q56JH9_DROME	0.38	0.58	0.67	0.91	0.8	0.88
tr:F7VJU3:F7VJU3_DROME	0.38	0.58	0.67	0.91	0.8	0.88
tr:Q56JH8:Q56JH8_DROME	0.38	0.58	0.67	0.91	0.8	0.88
tr:Q9Y1J9:Q9Y1J9_DROME	0.32	0.54	0.67	0.91	0.8	0.88
tr:Q56JI0:Q56JI0_DROME	0.24	0.41	0.67	0.91	0.8	0.88
tr:B7YZE7:B7YZE7_DROME	0.24	0.41	0.67	0.91	0.8	0.88
tr:A1Z9N8:A1Z9N8_DROME	0.24	0.41	0.67	0.91	0.8	0.88
tr:Q56JH7:Q56JH7_DROME	0.24	0.4	0.67	0.91	0.8	0.88
tr:B7YZE8:B7YZE8_DROME	0.24	0.4	0.67	0.91	0.8	0.88
tr:A1Z9N7:A1Z9N7_DROME	0.24	0.4	0.67	0.91	0.8	0.88

Ih (FBgn0263397) associated phenotypes

conditional, hypoactive, neurophysiology defective, optomotor response defective, proboscis extension reflex defective, short lived, sleep defective, uncoordinated

(Information from FlyBase)

Potassium/sodium hyperpolarization-activated cyclic nucleotide-gated channel 1

Best gene implication in ageing for this target family came from gene Q8IGX2 annotated in UniProt release 2014_02. Annotation GO 8340 (determination of adult lifespan) was Inferred from Mutant Phenotype

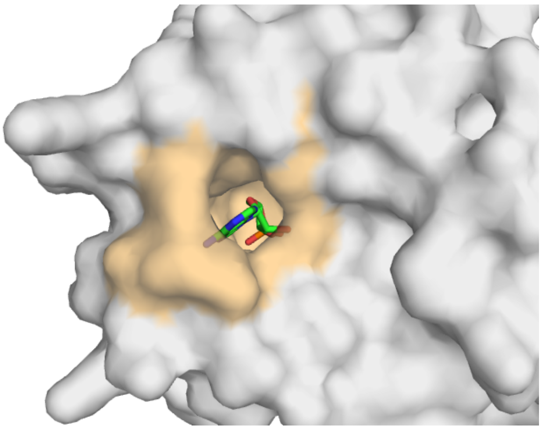

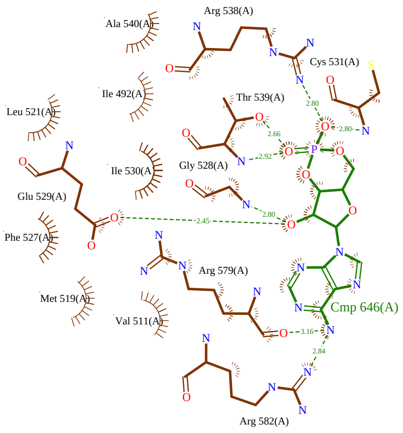

protein	amino acids contacts (binding site)
PDB:3u0z:chainA:O88704	I V M L F G E I C R T A R R
sp:Q9UL51:HCN2_HUMAN	I V M L F G E I C R T A R R
sp:O60741:HCN1_HUMAN	I V M L F G E I C R T A R R
tr:F1LRY7:F1LRY7_RAT	I V M L F G E I C R T A R R
tr:F1LSH6:F1LSH6_RAT	I V M L F G E I C R T A R R
sp:O88703:HCN2_MOUSE	I V M L F G E I C R T A R R
sp:O88704:HCN1_MOUSE	I V M L F G E I C R T A R R
tr:Q8IGX2:Q8IGX2_DROME	I V T L F G E I C R V A R K
tr:A1Z9P0:A1Z9P0_DROME	I V T L F G E I C R V A R K
tr:Q56JH9:Q56JH9_DROME	I V T L F G E I C R V A R K
tr:Q56JH8:Q56JH8_DROME	I V T L F G E I C R V A R K
tr:F7VJU3:F7VJU3_DROME	I V T L F G E I C R V A R K
tr:Q9Y1J9:Q9Y1J9_DROME	I V T L F G E I C R V A R K
tr:Q56JI0:Q56JI0_DROME	I V T L F G E I C R V A R K
tr:B7YZE7:B7YZE7_DROME	I V T L F G E I C R V A R K
tr:A1Z9N8:A1Z9N8_DROME	I V T L F G E I C R V A R K
tr:Q56JH7:Q56JH7_DROME	I V T L F G E I C R V A R K
tr:B7YZE8:B7YZE8_DROME	I V T L F G E I C R V A R K
tr:A1Z9N7:A1Z9N7_DROME	I V T L F G E I C R V A R K

	whole protein		domain-based		contact-based	
protein	ident	simil	ident	simil	ident	simil
PDB:3u0z:chainA:O88704	1.0	1.0	1.0	1.0	1.0	1.0
sp:Q9UL51:HCN2_HUMAN	0.54	0.71	0.8	0.9	1.0	1.0
sp:O60741:HCN1_HUMAN	0.92	0.94	0.98	1.0	1.0	1.0
tr:F1LRY7:F1LRY7_RAT	0.56	0.73	0.8	0.91	1.0	1.0
tr:F1LSH6:F1LSH6_RAT	0.98	0.99	1.0	1.0	1.0	1.0
sp:O88703:HCN2_MOUSE	0.55	0.71	0.8	0.91	1.0	1.0
sp:O88704:HCN1_MOUSE	1.0	1.0	1.0	1.0	1.0	1.0
tr:Q8IGX2:Q8IGX2_DROME	0.23	0.34	0.46	0.78	0.79	0.88
tr:A1Z9P0:A1Z9P0_DROME	0.34	0.54	0.46	0.78	0.79	0.88
tr:Q56JH9:Q56JH9_DROME	0.34	0.54	0.46	0.78	0.79	0.88
tr:Q56JH8:Q56JH8_DROME	0.34	0.54	0.46	0.78	0.79	0.88
tr:F7VJU3:F7VJU3_DROME	0.34	0.53	0.46	0.78	0.79	0.88
tr:Q9Y1J9:Q9Y1J9_DROME	0.27	0.44	0.46	0.78	0.79	0.88
tr:Q56JI0:Q56JI0_DROME	0.21	0.35	0.46	0.78	0.79	0.88
tr:B7YZE7:B7YZE7_DROME	0.21	0.34	0.46	0.78	0.79	0.88
tr:A1Z9N8:A1Z9N8_DROME	0.21	0.34	0.46	0.78	0.79	0.88
tr:Q56JH7:Q56JH7_DROME	0.21	0.34	0.46	0.78	0.79	0.88
tr:B7YZE8:B7YZE8_DROME	0.21	0.34	0.46	0.78	0.79	0.88
tr:A1Z9N7:A1Z9N7_DROME	0.21	0.34	0.46	0.78	0.79	0.88

Ih (FBgn0263397) associated phenotypes

conditional, hypoactive, neurophysiology defective, optomotor response defective, proboscis extension reflex defective, short lived, sleep defective, uncoordinated

(Information from FlyBase)

cAMP-dependent protein kinase regulatory subunit

Best gene implication in ageing for this target family came from gene P07278 via mapping the annotation from Ensembl YIL033C via mapping the annotation from EntrezGene 854778 via mapping the annotation from GenAgeModels 0042 annotated in GenAge release 17.

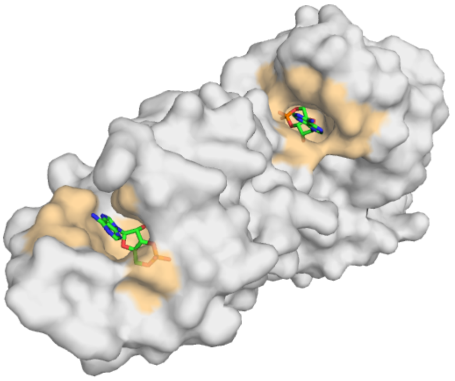

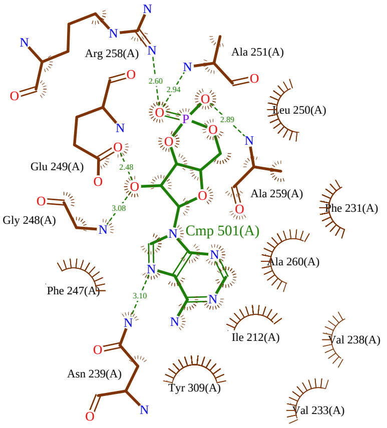

protein	amino acids contacts (binding site)																											
PDB:3of1:chainA:P07278	I	F	V	V	N	F	G	E	L	A	R	A	A	Y	I	V	I	N	F	G	E	V	A	R	Q	A	V	P
sp:P31323:KAP3_HUMAN	I	I	V	V	G	F	G	E	L	A	R	A	A	S	I	I	I	A	F	G	E	L	A	R	A	A	A	A
tr:R9PXT9:R9PXT9_RAT	I	I	V	V	G	F	G	E	L	A	R	A	A	S	I	I	I	A	F	G	E	L	A	R	A	A	A	A
tr:H3BK84:H3BK84_MOUSE	I	I	V	V	G	F	G	E	L	A	R	A	A	S	I	I	I	A	F	G	E	L	A	R	A	A	A	A
sp:P31324:KAP3_MOUSE	I	I	V	V	G	F	G	E	L	A	R	A	A	S	I	I	I	A	F	G	E	L	A	R	A	A	A	A
tr:A8DY88:A8DY88_DROME	I	V	I	I	N	F	G	E	L	A	R	A	A	Y	I	V	I	S	F	G	E	L	A	R	A	A	V	S
tr:A8DY87:A8DY87_DROME	I	V	I	I	N	F	G	E	L	A	R	A	A	Y	I	V	I	S	F	G	E	L	A	R	A	A	V	V
sp:P81900:KAPR2_DROME	I	V	I	I	N	F	G	E	L	A	R	A	A	Y	I	V	I	S	F	G	E	L	A	R	A	A	V	D
sp:P07278:KAPR_YEAST	I	F	V	V	N	F	G	E	L	A	R	A	A	Y	I	V	I	N	F	G	E	V	A	R	Q	A	V	P

	whole protein		domain-based		contact-based	
protein	ident	simil	ident	simil	ident	simil
PDB:3of1:chainA:P07278	1.0	1.0	1.0	1.0	1.0	1.0
sp:P31323:KAP3_HUMAN	0.3	0.66	0.36	0.7	0.68	0.8
tr:R9PXT9:R9PXT9_RAT	0.25	0.49	0.35	0.66	0.68	0.8
tr:H3BK84:H3BK84_MOUSE	0.21	0.4	0.32	0.59	0.68	0.8
sp:P31324:KAP3_MOUSE	0.3	0.66	0.36	0.7	0.68	0.8
tr:A8DY88:A8DY88_DROME	0.3	0.61	0.39	0.74	0.75	0.88
tr:A8DY87:A8DY87_DROME	0.31	0.62	0.4	0.76	0.75	0.89
sp:P81900:KAPR2_DROME	0.31	0.62	0.4	0.74	0.75	0.87
sp:P07278:KAPR_YEAST	1.0	1.0	1.0	1.0	1.0	1.0

Pka-R2 (FBgn0022382) associated phenotypes

chemical resistant, neuroanatomy defective

(Information from FlyBase)

Pka-R2 (UniProt:P81900) annotation

Function: Type II regulatory chains mediate membrane association by binding to anchoring proteins, including the MAP2 kinase. The regulatory subunit may play an essential role in the regulation of neuronal activity in the brain. Has a role in circadian locomotor rhythm, and behavioral response to cocaine and ethanol. (PubMed:10781603, PubMed:9196067).
Subunit: Tetramer, composed of 2 regulatory (R) and 2 catalytic (C) subunits. In the presence of cAMP it dissociates into 2 active monomeric C subunits and an R dimer (By similarity).
Subcellular location: Cytoplasm (PubMed:9196067).
Ptm: The pseudophosphorylation site binds to the substrate-binding region of the catalytic chain but is not phosphorylated. The physiological significance of phosphorylations by other kinases is unclear (By similarity).

(Information from UniProt)

Potassium/sodium hyperpolarization-activated cyclic nucleotide-gated channel 2

Best gene implication in ageing for this target family came from gene Q8IGX2 annotated in UniProt release 2014_02. Annotation GO 8340 (determination of adult lifespan) was Inferred from Mutant Phenotype

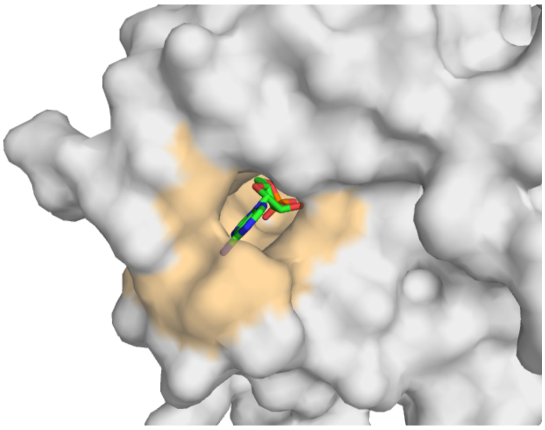

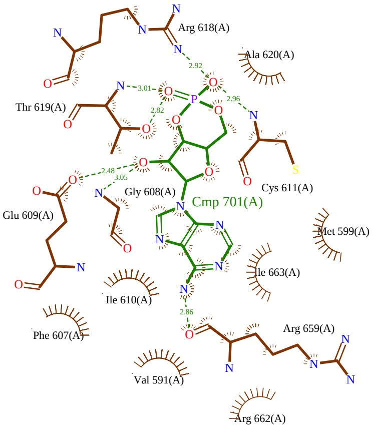

protein	amino acids contacts (binding site)
PDB:3u10:chainA:Q9UL51	V M F G E I C R T A R R I
sp:Q9UL51:HCN2_HUMAN	V M F G E I C R T A R R I
sp:O60741:HCN1_HUMAN	V M F G E I C R T A R R I
tr:F1LRY7:F1LRY7_RAT	V M F G E I C R T A R R I
tr:F1LSH6:F1LSH6_RAT	V M F G E I C R T A R R I
sp:O88703:HCN2_MOUSE	V M F G E I C R T A R R I
sp:O88704:HCN1_MOUSE	V M F G E I C R T A R R I
tr:Q8IGX2:Q8IGX2_DROME	V T F G E I C R V A R K I
tr:A1Z9P0:A1Z9P0_DROME	V T F G E I C R V A R K I
tr:Q56JH9:Q56JH9_DROME	V T F G E I C R V A R K I
tr:Q56JH8:Q56JH8_DROME	V T F G E I C R V A R K I
tr:F7VJU3:F7VJU3_DROME	V T F G E I C R V A R K I
tr:Q9Y1J9:Q9Y1J9_DROME	V T F G E I C R V A R K I
tr:Q56JI0:Q56JI0_DROME	V T F G E I C R V A R K I
tr:B7YZE7:B7YZE7_DROME	V T F G E I C R V A R K I
tr:A1Z9N8:A1Z9N8_DROME	V T F G E I C R V A R K I
tr:Q56JH7:Q56JH7_DROME	V T F G E I C R V A R K I
tr:B7YZE8:B7YZE8_DROME	V T F G E I C R V A R K I
tr:A1Z9N7:A1Z9N7_DROME	V T F G E I C R V A R K I

protein	whole protein ident	simil	domain-based ident	simil	contact-based ident	simil
PDB:3u10:chainA:Q9UL51	1.0	1.0	1.0	1.0	1.0	1.0
sp:Q9UL51:HCN2_HUMAN	1.0	1.0	1.0	1.0	1.0	1.0
sp:O60741:HCN1_HUMAN	0.57	0.74	0.91	0.98	1.0	1.0
tr:F1LRY7:F1LRY7_RAT	0.89	0.92	1.0	1.0	1.0	1.0
tr:F1LSH6:F1LSH6_RAT	0.55	0.71	0.91	0.98	1.0	1.0
sp:O88703:HCN2_MOUSE	0.91	0.95	1.0	1.0	1.0	1.0
sp:O88704:HCN1_MOUSE	0.54	0.71	0.91	0.98	1.0	1.0
tr:Q8IGX2:Q8IGX2_DROME	0.25	0.37	0.67	0.91	0.77	0.86
tr:A1Z9P0:A1Z9P0_DROME	0.37	0.56	0.67	0.91	0.77	0.86
tr:Q56JH9:Q56JH9_DROME	0.37	0.56	0.67	0.91	0.77	0.86
tr:Q56JH8:Q56JH8_DROME	0.37	0.56	0.67	0.91	0.77	0.86
tr:F7VJU3:F7VJU3_DROME	0.37	0.56	0.67	0.91	0.77	0.86
tr:Q9Y1J9:Q9Y1J9_DROME	0.32	0.55	0.67	0.91	0.77	0.86
tr:Q56JI0:Q56JI0_DROME	0.24	0.42	0.67	0.91	0.77	0.86
tr:B7YZE7:B7YZE7_DROME	0.24	0.42	0.67	0.91	0.77	0.86
tr:A1Z9N8:A1Z9N8_DROME	0.24	0.42	0.67	0.91	0.77	0.86
tr:Q56JH7:Q56JH7_DROME	0.24	0.41	0.67	0.91	0.77	0.86
tr:B7YZE8:B7YZE8_DROME	0.24	0.41	0.67	0.91	0.77	0.86
tr:A1Z9N7:A1Z9N7_DROME	0.24	0.41	0.67	0.91	0.77	0.86

Ih (FBgn0263397) associated phenotypes

conditional, hypoactive, neurophysiology defective, optomotor response defective, proboscis extension reflex defective, short lived, sleep defective, uncoordinated

(Information from FlyBase)

Potassium/sodium hyperpolarization-activated cyclic nucleotide-gated channel 4

Best gene implication in ageing for this target family came from gene Q8IGX2 annotated in UniProt release 2014_02. Annotation GO 8340 (determination of adult lifespan) was Inferred from Mutant Phenotype

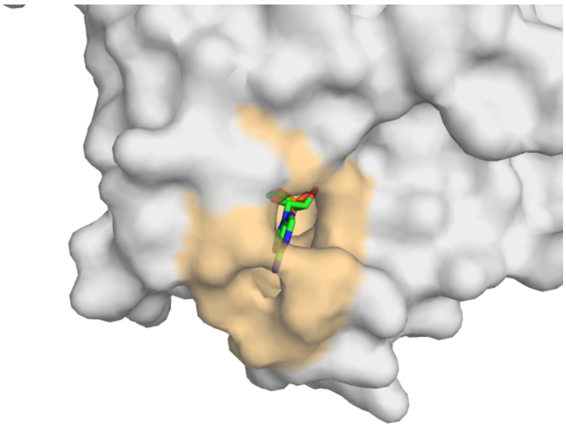

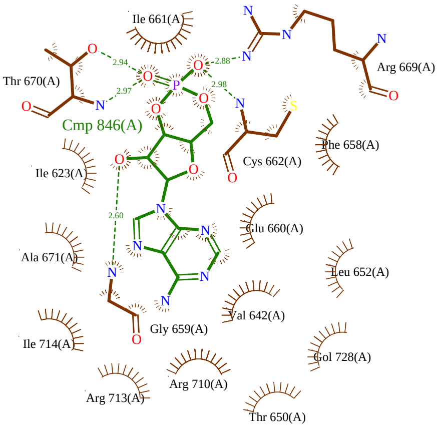

protein	amino acids contacts (binding site)
PDB:3otf:chainA:Q9Y3Q4	I V E T L F G E I C R T A R R I
PDB:3u11:chainA:Q9Y3Q4	I V E T L F G E I C R T A R R I
PDB:4hbn:chainA:Q9Y3Q4	I V E T L F G E I C R T A R R I
sp:Q9Y3Q4:HCN4_HUMAN	I V E T L F G E I C R T A R R I
sp:Q9JKA7:HCN4_RAT	I V E T L F G E I C R T A R R I
tr:B2RY58:B2RY58_MOUSE	I V E T L F G E I C R T A R R I
tr:Q8IGX2:Q8IGX2_DROME	I V A T L F G E I C R V A R K I
tr:A1Z9P0:A1Z9P0_DROME	I V A T L F G E I C R V A R K I
tr:Q56JH9:Q56JH9_DROME	I V A T L F G E I C R V A R K I
tr:Q56JH8:Q56JH8_DROME	I V A T L F G E I C R V A R K I
tr:F7VJU3:F7VJU3_DROME	I V A T L F G E I C R V A R K I
tr:Q9Y1J9:Q9Y1J9_DROME	I V A T L F G E I C R V A R K I
tr:Q56JI0:Q56JI0_DROME	I V A T L F G E I C R V A R K I
tr:B7YZE7:B7YZE7_DROME	I V A T L F G E I C R V A R K I
tr:A1Z9N8:A1Z9N8_DROME	I V A T L F G E I C R V A R K I
tr:Q56JH7:Q56JH7_DROME	I V A T L F G E I C R V A R K I
tr:B7YZE8:B7YZE8_DROME	I V A T L F G E I C R V A R K I
tr:A1Z9N7:A1Z9N7_DROME	I V A T L F G E I C R V A R K I

	whole protein		domain-based		contact-based	
protein	ident	simil	ident	simil	ident	simil
PDB:3otf:chainA:Q9Y3Q4	1.0	1.0	1.0	1.0	1.0	1.0
PDB:3u11:chainA:Q9Y3Q4	0.99	1.0	0.99	0.99	1.0	1.0
PDB:4hbn:chainA:Q9Y3Q4	0.99	0.99	0.99	1.0	1.0	1.0
sp:Q9Y3Q4:HCN4_HUMAN	1.0	1.0	1.0	1.0	1.0	1.0
sp:Q9JKA7:HCN4_RAT	0.96	0.98	1.0	1.0	1.0	1.0
tr:B2RY58:B2RY58_MOUSE	0.95	0.98	1.0	1.0	1.0	1.0
tr:Q8IGX2:Q8IGX2_DROME	0.19	0.27	0.68	0.91	0.81	0.88
tr:A1Z9P0:A1Z9P0_DROME	0.28	0.41	0.68	0.91	0.81	0.88
tr:Q56JH9:Q56JH9_DROME	0.27	0.41	0.68	0.91	0.81	0.88
tr:Q56JH8:Q56JH8_DROME	0.27	0.41	0.68	0.91	0.81	0.88
tr:F7VJU3:F7VJU3_DROME	0.27	0.41	0.68	0.91	0.81	0.88
tr:Q9Y1J9:Q9Y1J9_DROME	0.26	0.47	0.68	0.91	0.81	0.88
tr:Q56JI0:Q56JI0_DROME	0.21	0.38	0.68	0.91	0.81	0.88
tr:B7YZE7:B7YZE7_DROME	0.21	0.37	0.68	0.91	0.81	0.88
tr:A1Z9N8:A1Z9N8_DROME	0.21	0.37	0.68	0.91	0.81	0.88
tr:Q56JH7:Q56JH7_DROME	0.21	0.37	0.68	0.91	0.81	0.88
tr:B7YZE8:B7YZE8_DROME	0.21	0.37	0.68	0.91	0.81	0.88
tr:A1Z9N7:A1Z9N7_DROME	0.21	0.37	0.68	0.91	0.81	0.88

Ih (FBgn0263397) associated phenotypes

conditional, hypoactive, neurophysiology defective, optomotor response defective, proboscis extension reflex defective, short lived, sleep defective, uncoordinated

(Information from FlyBase)
